# Regulation of B cell development and lymphocyte function by transcriptional coactivator OCA-B

**DOI:** 10.3389/fimmu.2026.1764360

**Published:** 2026-03-19

**Authors:** Xiangdong Lu, Robert G. Roeder

**Affiliations:** Laboratory of Biochemistry and Molecular Biology, The Rockefeller University, New York, NY, United States

**Keywords:** B cell development, BCL6, coactivator, follicular T helper cells, germinal center, memory T cell, OCA-B, transcriptional regulation

## Abstract

OCA-B (OCT Coactivator from B cells) is highly expressed in B cells and is the first-identified tissue-specific transcriptional coactivator. Mechanistically, OCA-B is recruited to target genes through primary interactions with DNA-binding transcription factors OCT1/OCT2, as well as secondary interactions with MEF2B, and its effector functions in activating transcription involve interactions with the Mediator coactivator complex. Physiologically, OCA-B plays an essential role in antigen-stimulated Germinal Center (GC) formation and transcription of secondary immunoglobulin (Ig) genes. OCA-B also regulates bone marrow and peripheral B cell development. While GC B cell-specific inactivation of *Oca-B* is sufficient to cause GC defects, OCA-B function in follicular T helper (Tfh) cells also plays an important role in GC reactions. In GC B cells, OCA-B–dependent genes include known GC regulatory genes such as *Bcl6*, *Mef2b* and *Irf4*. In CD4^+^ memory T cells, OCA-B is required to maintain a subset of genes, including *Il2*, in a transcriptionally poised state, enabling their rapid and robust up-regulation upon repeated stimulation. Moreover, recent studies have indicated an essential role for OCA-B in B-cell malignancy and multiple autoimmune diseases, highlighting OCA-B as a potential therapeutic target for these diseases.

## Introduction

Transcription is regulated by DNA binding transcription factors (TFs) that act through various cofactors to enhance or repress the function of the basal transcription machinery, including RNA polymerase II (Pol II) and cognate general initiation factors (GTFs). These cofactors comprise both chromatin-modifying factors (e.g., the p300/CBP histone acetyltransferases and the SET1/MLL H3K4 methyltransferases) and other factors (e.g., Mediator, TAFs) that facilitate more direct communication between enhancer-bound regulatory factors and the general transcription machinery at the promoter (1). A central question in transcriptional regulation is how distinct cell types, despite sharing identical genomic sequences, establish unique gene expression programs. The B cell-restricted transcription of immunoglobulin (Ig) genes served as one important initial model for investigating the mechanisms controlling cell type-specific gene expression. In addition to the crucial TATA box in the core promoter, nearly all Ig gene promoters contain an essential upstream octamer motif (5´ATGCAAAT 3´ or its inversion) (2). As one of the initial pieces of evidence for the cell-specific regulatory role of the octamer motif, synthetic reporters combining only octamer and TATA motifs proved sufficient for B cell-specific expression and were only active in lymphocytes (3). As an important extension and confirmation of these analyses, transgenic mouse studies revealed that mutation of the promoter-associated octamer motif significantly reduced expression of the Igh locus in lymphocytes (4). These results thus demonstrated a critical role for the octamer motif in B-cell–specific Ig gene expression and prompted studies to identify related transcription factors. POU domain-containing transcription factors, including the ubiquitously expressed OCT1 and the B cell-restricted OCT2, were subsequently identified as octamer-binding factors – and an initial model proposed that OCT2 alone was responsible for B cell-specific Ig gene transcription (5, 6). However, this view was challenged by findings that Ig genes are still highly expressed in OCT2-deficient B cells (6). This paradox was partially resolved with the discovery of OCA-B (also known as OBF1 or BOB1 and encoded by the *POU2AF1* gene), the prototype tissue-specific transcriptional coactivator, more than three decades ago (7).

The *Oca-B* gene is highly expressed in bone marrow and peripheral B cells, plasma cells, and follicular T helper cells – and is also expressed in activated (but not naïve) CD4^+^ T cells (6, 8, 9). OCA-B can significantly enhance OCT2- or OCT1-mediated transcription *in vitro* or in cells (5, 10). Soon after the discovery of OCA-B and the cloning of the *OCA-B* gene, several laboratories independently generated *Oca-B* knockout mice to investigate the function of OCA-B *in vivo*. Surprisingly, the most severe defects of OCA-B null mice were the loss of germinal center formation and the production of secondary immunoglobulin isotypes in antigen-stimulated immune responses, with early B cell development in the bone marrow and the production of IgM seemingly largely unaffected (11–13). The findings of the early OCA-B studies have been described in earlier published reviews (5, 6, 10). Here, we summarize some of those findings along with more recent studies on the mechanism of action of OCA-B, the function of OCA-B in B cell development and the germinal center reactions, and OCA-B target genes in activated B cells. We also review the more recently discovered function of OCA-B in follicular T helper cells, CD4^+^ T cell memory, and the function of OCA-B in B cell lymphomagenesis and autoimmune diseases.

## OCA-B as a transcriptional coactivator

OCA-B is a proline-rich protein with 34 and 35 KDa isoforms encoded by the same gene. As a coactivator, OCA-B does not bind to specific DNA motifs and cannot activate transcription on its own; instead, it enhances OCT1- and OCT2-mediated transcription as first demonstrated for Ig genes (10). The POU domain of OCT1 (or OCT2) is composed of two structurally independent DNA-binding domains, the POU-specific domain (POU_S_) that contacts the 5’ half of the octamer motif and the POU homeodomain (POU_H_) that contacts the 3’ half of the octamer motif (14). Previous structural and mutational analyses have shown that a region of seven amino acids (amino acids 26-32) in the N-terminal region of OCA-B interacts with the highly related POU domains of OCT1 and OCT2, thereby enhancing their binding to octamer motifs at Ig gene promoters and enhancers by clamping the POU_S_ and POU_H_ subdomains (6, 10). However, OCA-B is specific for OCT1 and OCT2 and does not interact with the POU domains of OCT3, OCT6 and OCT11 (5). Structural analyses of the OCT1–OCA-B–octamer motif ternary complex and mutational studies have shown that this selective OCA-B binding to OCT1/2 is determined by a few residues in the POUs and POU_H_ domains of OCT1/2 (15, 16). A recent study also showed that OCT2 and OCA-B mutants carrying mutations at these residues lose their function in lymphoma cells, confirming that OCA-B activates transcription through OCT1/2 (17). Previous studies have also shown that a stretch of acidic amino acids very close to the C-terminus displays a transcription activation function. Mutation of this region, although not abrogating OCA-B binding to the OCT1-DNA complex, impairs the coactivation function of OCA-B *in vitro* and in cell-based assays, although the mechanism of this apparent activation domain is not yet established (10). In addition to POU domain binding specificity, OCA-B also shows selective octamer interactions. A biochemical analysis showed that OCA-B only binds octamers that have adenine at position 5 (A_5_) and contacts DNA in the major groove through direct interaction with A_5_ of the octamer motif (10). The physiological relevance of this observation is evidenced by the fact that *de novo* motif analysis of chromatin immunoprecipitation-sequencing (ChIP-seq) data from a GC-derived lymphoma cell line and *in vitro* stimulated primary murine splenic B cells identified 5’-ATGCAAAT-3’, which matches the consensus octamer motif, as a top enriched motif in OCA-B peaks (18–20).

**Figure 1:**
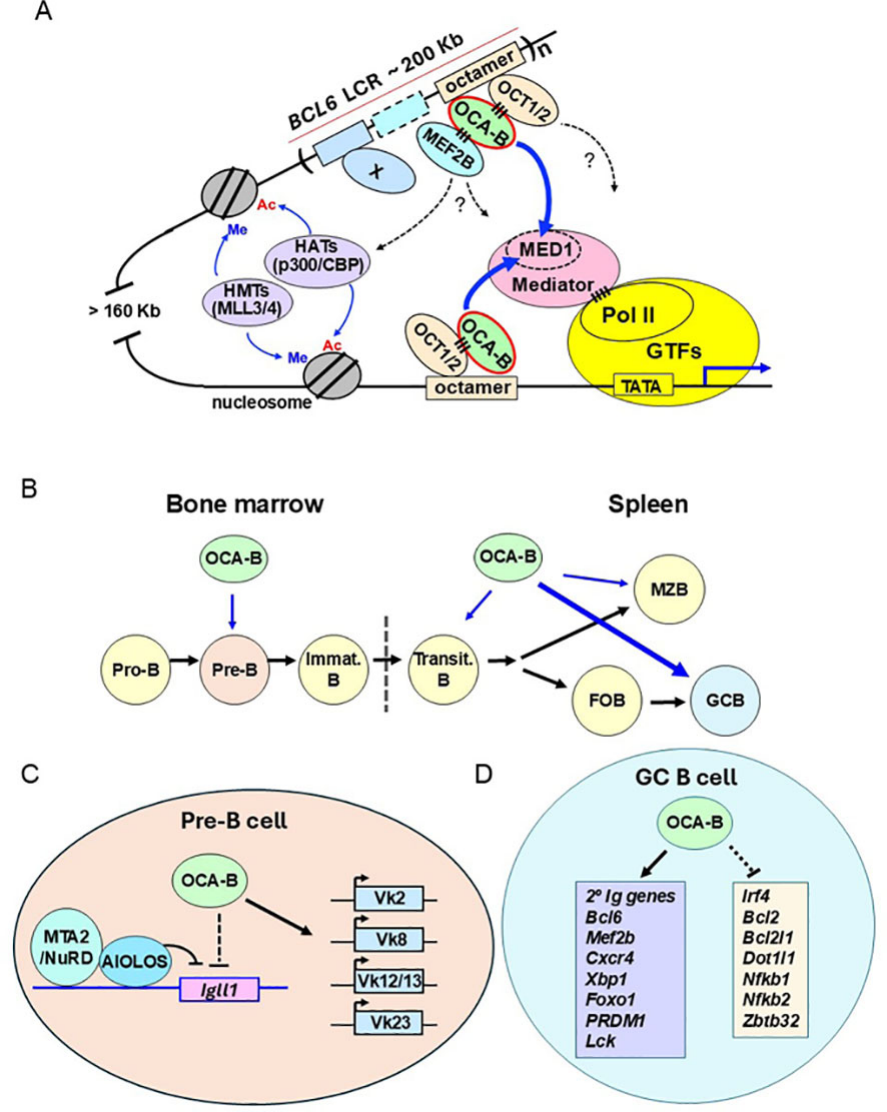
The mechanism and function of OCA-B-dependent transcriptional regulation in B cell development. **(A)** OCA-B–dependent transcriptional activation of the *BCL6* gene. The figure indicates DNA-binding transcription factors (OCT1/2, MEF2B) and interacting transcriptional co-activators (MED1/Mediator) that have been localized to the human *BCL6* Locus Control Region (LCR)/super enhancer and shown to interact directly as indicated. For details, see text and reference (18). Note that whereas MEF2B can act as a site-specific DNA binding transcription factor, the formation and function of the ternary OCT1/2–OCA-B–MEF2B complex on the *BCL6* DNA octamer regulatory element is independent of the DNA binding activity of MEF2B–which in this case acts as a secondary transcriptional coactivator. Other transcription factors (designated by X) are also known to facilitate *BCL6* LCR function. Dashed arrows indicate as yet undetermined interactions of activation domains of OCT1/2 and MEF2B, although a direct MEF2-p300 interaction has been demonstrated (18). HAT, histone acetyltransferase; HMT, histone methyltransferase; Ac, histone acetylation; Me, H3K4 methylation. Note that whereas the model shown is based on recent studies of the *BCL6* gene (18), the basic principles are also applicable to other B cell-specific genes such as the Ig genes that also contain regulatory octamer elements at both promoter proximal and distal enhancer sites and that were originally shown to be regulated by OCA-B through OCT1/2 interactions. **(B)** Schematic diagram of the role of OCA-B in B cell development and function at different B cell developmental stages. OCA-B plays an essential role in regulating germinal center reactions (thick arrow). OCA-B also plays important roles in pre-B, transitional B, and marginal zone B cells (thin arrows). **(C)** OCA-B both activates a group of *Igκ* genes and cooperates with Aiolos and the Mta2/NuRD complex to repress the *Igll1* gene in pre-B cells. **(D)** Representative genes activated and repressed by OCA-B in germinal center B cells. 2^°^ Ig genes indicate secondary immunoglobulin genes. For further details and references see text.

Early biochemical studies showed that OCA-B–OCT1/2 synergy requires the general positive cofactor PC4 and the Mediator coactivator complex (10). OCA-B was also found to mediate long-range *Igh* enhancer-promoter interactions at the *Igh* locus through interactions between enhancer-bound OCT1/2–OCA-B and promoter-bound TFII-I (21). More recently, OCA-B was found to form a ternary complex with OCT2 and MEF2b at the *BCL6* Locus Control Region (LCR), thereby regulating *BCL6* gene expression in B cell lymphoma cells (**Figure 1A**) (18). Beyond biochemical analyses showing direct interactions indicating a DNA→OCT1/2→OCA-B→MEF2B assembly pathway, combined CRISPR-based knockout and ChIP-seq analyses showed a highly cooperative chromatin binding of OCT1/2, OCA-B, and MEF2B in cells. It was further shown that OCA-B directly interacts with MED1 to recruit the Mediator complex to the *BCL6* LCR to facilitate long range (~ 160Kb) enhancer-promoter interactions (**Figure 1A****).** In the same study, a CRISPR interference (CRISPRi) analysis of the *BCL6* Locus Control Region (LCR) in lymphoma cells showed that deletion of octamer-containing elements in the *BCL6* LCR markedly decreased *BCL6* expression and inhibited lymphoma cell growth (18). This result is consistent with previous findings of a regulatory role for the octamer motif in B cell-specific expression of certain Ig genes.

In addition to the above-described interactions, OCA-B has been shown to interact with the histone3 lysine9 (H3K9) demethylase Jmjd1a in activated CD4^+^ T cells, the first indication of an OCA-B interaction with a chromatin-modifying coactivator. This interaction is required for robust induction of the *Il2* gene in restimulated memory T cells (9). A detailed analysis showed that the OCA-B–Jmjd1a interaction is mediated by the N-terminal 30–38 residues of OCA-B, and that mutations of lysine 31 and arginine 34 can abolish this interaction (22). Two more recent studies have indicated association of OCA-B with the transcription factor IRF4 and the SWI/SNF chromatin remodeling complex (23, 24). In the first study, IRF4 and multiple components of the SWI/SNF complex were identified as OCA-B–associated proteins by the rapid immunoprecipitation mass spectrometry of endogenous proteins (RIME) approach in multiple myeloma (MM) cells (23). ChIP-seq data from this study further showed genome-wide colocalization of IRF4 and OCA-B in MM cells, indicating that OCA-B might potentially act as a coactivator for IRF4. Consistent with the RIME results, the chromatin binding of OCA-B and IRF4 was found to depend on SWI/SNF activity (23). In the second study, more than 60 proteins that include components of the SWI/SNF complex and the H3K9 demethylase Jmjd1c were identified as potential OCA-B–associated factors by a proximity labeling approach in T cells (24). Since proximity labeling and RIME methods can identify proteins that either indirectly or transiently interact with OCA-B, further experiments are needed to distinguish direct and indirect OCA-B interacting factors.

In summary, these findings support a model in which (i) OCA-B is first recruited to DNA regulatory (octamer) elements through primary interactions with octamer-bound OCT1/OCT2, with concomitant stabilization of OCT1/2 DNA binding (along with MEF2B recruitment in germinal center B cells) and (ii) OCA-B subsequently exerts its downstream effector function through recruitment of other factors such as Mediator, TFII-I, and Jmjd1a to facilitate target gene activation – with the SWI/SNF complex facilitating interactions of these factors with chromatin sites.

## OCA-B isoforms and paralogs

OCA-B was the first-discovered tissue-specific transcription coactivator. As mentioned earlier, the *Oca-B* gene is highly expressed in B cells, plasma cells, and follicular T helper cells – and is also expressed in activated (but not naïve) CD4^+^ T cells. The *Oca-B* gene generates a single mRNA transcript that encodes two protein isoforms with molecular weights of 34 and 35 KDa (p34 and p35, respectively) (25). The p35 isoform is derived from a transient 40 KDa precursor (p40) that is translated from an in frame initiation codon that lies upstream of the p34 initiation codon and is subsequently processed post-translationally (26). It has also been shown that p35 undergoes N-terminal myristoylation, which leads to localization of p35 to the cellular membrane, suggesting a potentially important function of p35 in signal transduction in B cells (26). *In vitro* transcription experiments showed that p34 and p35 can independently stimulate the octamer-containing *Igh* promoter to comparable levels (25). However, while p34 can robustly induce expression of a reporter gene driven by an octamer-containing promoter, p35 fails to do so. This discrepancy between data from *in vitro* and cell-based assays is likely a result of the intracellular localization of p35. A following study showed, surprisingly, that the p35 OCA-B isoform directly interacts with SYK, a tyrosine kinase critical for pre-BCR and BCR signaling, in the cytoplasm and stabilizes the SYK protein (27). This result suggests an important role for OCA-B in regulating SYK-mediated signaling pathways during B cell development and function. Since both OCA-B isoforms are depleted in currently used *Oca-B* knockout mouse strains, further investigation is needed to elucidate which of the known functions of OCA-B in B cells and T cells described below are contributed by cytoplasmic OCA-B.

A recent study has identified two OCA-B paralogs, designated OCA-T1 (POU2AF2) and OCA-T2 (POU2AF3), and corresponding genes reside in a gene cluster on chromosome 11 in the human genome (28). OCA-T1 and OCA-T2 are found in tuft cells (chemosensory cells within mucosal epithelial tissues) and in tuft cell-like human tumor cells (a variant of small-cell lung cancer cells) and interact with octamer-binding transcription factor OCT11 (POU2F3) in these cells. Similar to the OCA-B–OCT1/2–octamer motif interaction, OCA-T1 and OCA-T2 bind to OCT11 and the octamer motif through a conserved peptide. Further consistent with previous results from the OCA-B studies, the binding of OCA-T1 to OCT11 and DNA requires adenine as the fifth base of the octamer (28). The OCA-T1/OCT11 or OCA-T2/OCT11 complex was further shown to drive expression of tuft cell-specific genes and to play an essential role in normal and neoplastic tuft cell growth (28–30). Currently, there are no reports on the expression and function of *OCA-T1* and *OCA-T2* in B cells and T cells.

## Emergence of the *Oca-B* gene during evolution of the adaptive immune system

While the innate immune system appears to exist in every multicellular organism (31), the adaptive immune system (AIS) only began to emerge in early vertebrates around 500 million years ago (32, 33). The most evolutionary ancient organisms that have components of AIS such as *TCR*, *BCR*, *RAG1/RAG2* are jawed fish. Regarding the origin of the *OCA-B* gene, in addition to mammals, *OCA-B* orthologs have been identified in birds, reptiles and amphibians, bony fish and cartilaginous fish. Recent studies also identified non-RAG–based AIS in jawless fish – namely lampreys. Related, orthologs of many transcription factor genes involved in lymphocyte development have been identified in lamprey and include *SPI-B, IKAROS*, *EBF1*, *GATA*, *PAX*-2/5/8 and *BACH2* (33, 34). While no *OCA-B* ortholog has been found in lamprey, orthologs have been found in both bony fish (Teleosts) such as salmon and catfish and in cartilaginous fish such as shark. A functional study of the channel catfish (*Ictalurus punctatus*) *OCA-B* ortholog showed that the POU binding region of the catfish OCA-B is 95% identical at the amino acid level with the binding region of human OCA-B, and all the residues directly involved in binding to the OCT-DNA complex are conserved (35). Despite this conservation, catfish OCA-B failed to enhance catfish OCT2-dependent transcriptional activation in a catfish cell line in reporter assays, apparently due to the lack of a functional activation domain within the catfish OCA-B (35). Based on what is known about human OCA-B–dependent transcription activation, it will be interesting to know whether catfish OCA-B, unlike human OCA-B, cannot interact with the catfish Mediator complex through MED1. In summary, during evolution, the *OCA-B* gene arose in jawed fish together with other key AIS genes, such as the *Rag1/2*, immunoglobulin, and *TCR* genes, to form complex adaptive immune systems in these organisms.

## The function of OCA-B in B cell development

Since early *in vitro* transcription assays and cell-based experiments showed a crucial role for OCA-B in OCT1- or OCT2-mediated Ig gene promoter activation, mice lacking OCA-B activity were expected to show severe defects in early B cell development and impaired IgM expression. However, the initial analysis of *Oca-B* null mice showed largely normal bone marrow (BM) B cell development and normal serum IgM levels, except for reduction of recirculating B cells and total splenic B cells (**Figure 1B****) (**6, 11–13). These results indicated that OCA-B is dispensable for activation of the Ig heavy chain (IgH) constant mu gene (*Ighm*). The most striking defects in *Oca-B* null mice are the loss of germinal center formation and a deficiency in production of secondary immunoglobulin isotypes that is caused by reduction in transcription of corresponding switched IgH constant region genes (6). Surprisingly, a subsequent genetic analysis showed that in mice with the lymphoid compartment reconstituted from fetal liver cells lacking both OCA-B and OCT2, the serum IgM level is normal and BM B cell development is largely unaffected until the immature B stage (36). These data suggest a functional redundancy between OCT2/OCA-B and OCT1 in activation of the *Ighm* gene during BM B cell development. The reduction of splenic B cells in *Oca-B* null mice is likely caused by a decreased number of splenic transitional B cells, which might be due to increased apoptosis of bone marrow transitional B cells (B220^+^IgM^hi^) and defective splenic seeding (6). Separate studies in mice lacking Bruton’s tyrosine kinase (BTK), a key component of the signaling pathway downstream of the BCR, had shown largely normal BM B cell development and a severely reduced mature B cell compartment (37–39) -- indicating BTK functions downstream of OCA-B during B cell development. A subsequent genetic analysis of mice lacking both *Oca-B* and *Btk* genes demonstrated that while the double knockout mice show a largely normal pre-B compartment and a 2-3–fold reduction in IgM^+^ B cells in bone marrow, they have lost nearly all B cells in the spleen and lymph nodes. Consistent with these results, the serum Ig levels, including the IgM level that is normal in *Oca-B* null mice, were either extremely low or below the detection limit in *Oca-B, Btk* double null mice (40). These data suggest that OCA-B and BTK control the mature B cell population in two different pathways. It is also worth noting that the Ig phenotype of double mutant mice is strikingly similar to the symptoms in human X-linked agammaglobulinemia (XLA) patients (41). In the human clinic, *BTK* gene mutations have been found in about 90% of males with presumed XLA (42). Interestingly, it recently was reported that an agammaglobulinemia patient shows a homozygous *OCA-B* gene mutation (frameshift mutation) (43), which is the first case linking OCA-B deficiency to human immunodeficiency disease.

Although *Oca-B* null mice exhibit normal IgM production during BM B cell development (6, 10), subsequent analyses of Ig light chain gene rearrangements demonstrated that OCA-B plays a critical role in the transcription and recombination of a subset of *Vκ* genes at the pre-B stage (**Figure 1C**) (44). The differential OCA-B dependencies of *Ighm* and *Igκ* genes may reflect sequence differences between octamer motifs of these loci (44, 45). In addition, distinct sets of transcriptional regulatory factors binding to these genes may further modulate their OCA-B requirements. As discussed below, differences in octamer motif sequences may also contribute to the differential OCA-B dependencies of the *Igh* intronic enhancer (Eμ) and the 3’ enhancer, which in turn regulate the expression of *Ighm* and switched *Igh* isotype genes.

After rearrangement of Ig heavy chain genes at the pro-B (pre-BI) stage (B220^+^IgM^-^CD43^+^), cells progress to the pre-B (pre-BII) stage (B220^+^IgM^-^CD43^-^CD25^+^). At this stage, the heavy chain pairs with the surrogate light chain (SLC) to form the pre-B cell receptor (pre-BCR). The pre-BCR with a functional heavy chain mediates signaling to induce cell proliferation and survival, progressing through the first checkpoint during B cell development. The *Igll1* and *VpreB1/VpreB2* genes that, respectively, encode the λ5 and VpreB subunits of the surrogate light chain are then downregulated and the pre-B cells exit from the cell cycle with subsequent Ig light chain *kappa* or *lambda* gene rearrangement. Two genetic analyses had revealed a key role of OCA-B in silencing surrogate light chain genes and controlling the pre-B to immature B transition (**Figure 1C**). In one study, *Oca-B* KO mice were crossed to *Aiolos* KO mice. Aiolos (IKZF3) is a member of the Ikaros (IKZF1) lymphocyte-specific transcription factor family and an important regulator of T cell and B cell function (46–48). While mice lacking either *Oca-B* or *Aiolos* show a mildly impaired or largely normal early B cell development, mice lacking both *Oca-B* and *Aiolos* genes fail to silence *Igll1* and *VpreB1* genes in pre-B cells – and also show impaired nuclear positioning of the *Igll1* gene, defective light chain gene rearrangement, and a severe pre-B to immature B transition block (49).

In another study, *Oca-B* null mice were crossed to mice lacking the *Mta2* gene, which encodes a subunit of the repressive Nucleosome Remodeling and Histone Deacetylase (NuRD) complex. While *Mta2*-deficient mice show an approximate 2-fold reduction of immature B cells, *Oca-B, Mta2* double mutant mice show a striking 10-fold reduction in immature B cells, and more than a 30-fold reduction in recirculating B cells in the BM (50). Furthermore, although either *Mta2* inactivation or *Oca-B* inactivation can cause inefficient silencing of the *Igll1* and *VpreB1* genes, the expression levels of these two genes are much higher in double mutant pre-B cells than in single mutant pre-B cells. In terms of splenic B cells, loss of Mta2 activity does not affect follicular B cells but causes a more than two-fold increase in marginal zone (MZ) cells. However, the joint loss of *Mta2* and *Oca-B* genes leads to 13-fold and 15-fold reductions, respectively, in follicular B cell and MZ B cell populations (50). These defects are very similar to those in *Oca-B*, *Aiolos* double knockout mice. Because Aiolos, like its paralog Ikaros, is directly associated with the NuRD complex in both T and B cells, these data strongly suggest a model in which OCA-B collaborates with the Aiolos/NuRD complex to control surrogate light chain silencing and the pre-B to immature B transition (**Figure 1C**).

In mice and humans, peripheral naïve B cells include three subsets: follicular B cells, marginal zone B cells and B-1 B cells. Mature follicular B cells recirculate between lymphoid tissues and mediate either T cell-dependent or T cell-independent immune responses. MZ B cells are located at the marginal zone of the spleen. B-1B cells mainly localize in peritoneal and pleural cavities. B-1 cells can be further divided into B-1a (CD5^+^) and B-1b (CD5^-^) cells. Both B-1 and MZ B cells are innate-like cells that mediate T-independent immune responses, providing first line protection against blood-borne pathogens (51). The effect of OCA-B inactivation on MZ B cell development is genetic background-dependent. While C57BL/6 *Oca-B* null mice show a significant loss of MZ B cells, C57BL/6 X 129Sv *Oca-B* null mice only show a modest MZ B cell defect (6). Furthermore, *Oca-B*–deficient MZ B cells show functional defects such as reduced MZ-specific antigen capture and BLC (B lymphocyte chemoattractant)-induced B cell migration (52). While B-1 B cell development in *Oca-B* knockout mice is largely normal, the mice lacking both *Oca-B* and *Nfkb1* genes show a much more severe B-1 B cell defect than do *Nfkb*1 KO mice. These results suggest complementary roles of these two factors in regulating B-1 B cell development (53).

## The function of OCA-B in germinal center reactions

Germinal centers (GCs) are microanatomical structures formed in secondary lymphoid organs during antigen-stimulated immune responses. GCs are sites where B cells undergo clonal expansion, somatic hypermutation, affinity-based selection, and differentiation into plasma cells and memory B cells and form an important part of humoral immunity (54, 55). The most severe defects in *Oca-B* null mice are the lack of GC formation and production of secondary immunoglobulin isotypes that include IgG1, IgG2a, IgG2b, IgG3, IgA and IgE. This secondary Ig deficiency is not due to a failure of isotype switching processes but, rather, is caused by reduced transcription from normally switched Ig heavy chain constant region genes (secondary Ig genes) (**Figure 1D**) (6). Mechanistically, it is known that activation of Ig heavy chain genes is controlled by the *Igh* intronic enhancer Eμ and the 3’ enhancer (also known as the *Igh* 3’ regulatory region, 3’RR) during B cell development and immune responses (56). Analysis of B cells in mice lacking Eμ demonstrated that Eμ plays a dominant role in *Ighm* expression and BM B cell development, with little or no effect on production of secondary antibodies during immune responses (57). On the other hand, mice lacking the *Igh* 3’ enhancer show normal BM B cell development but severe defects in Ig class switching and secretion of Ig isotypes accompanied by decreased levels of transcripts of all *Igh* isotype genes but, especially, those of *Igh γ2b*, *γ3*, *ε*, and *α* genes (58, 59). In this regard, it has also been shown that OCA-B and OCT2 are both required for *Igh* 3’ enhancer activity, which, as mentioned, controls the transcription of class-switched *Igh* genes (60, 61). As discussed earlier, different octamer motif sequences in *Vκ* gene promoters can affect their OCA-B dependencies (44). Altogether, these results suggest a model in which distinct octamer motif sequences within the Eμ and *Igh* 3′ enhancers, together with other transcriptional regulators bound to these elements, confer differential OCA-B requirements, resulting in OCA-B–independent Eμ–driven *Ighm* expression and OCA-B–dependent *Igh* 3’ enhancer–driven expression of switched *Igh* isotype genes.

To determine if the loss of GC formation and other B cell development defects in *Oca-B* null mice are cell-autonomous effects, a recent study used three *Cre* lines to inactivate the *Oca-B* gene at different B cell stages. The data show (i) that deletion of *Oca-B* at the pro-B stage (by *Cd19Cre*) recapitulates defects in BM B cells, follicular B and MZ B cells, and GC B cells observed in *Oca-B* null mice and (ii) that deletion of *Oca-B* at the mature B stage (by *Cd23Cre*) recapitulates defects in follicular B, MZ B, and GC B cells of *Oca-B* null mice. More importantly, deletion of *Oca-B* in GC B cells by *Cγ-1Cre* is sufficient to cause severe GC defects – suggesting a crucial GC B cell-intrinsic function of OCA-B during immune responses (62).

One key issue in understanding how OCA-B regulates GC reactions is to identify OCA-B–dependent genes, including both direct and indirect (downstream) targets, in GC B cells. Beyond the switched Ig heavy chain genes described above, one early study used a cDNA microarray approach to identify OCA-B–dependent genes in *in vitro* stimulated primary murine splenic B cells (63). Results from this study showed that the induction of *Lck*, *Cdc37*, *CyclinD3*, *Kcnn4* and *S100a10* genes in *Oca-B KO* cells is impaired in response to BCR ligation, and that the induction of *B4galt1* and *Ms4a11* is reduced in response to BCR and T helper cell stimulation. Although this initial study did not distinguish direct versus indirect OCA-B target genes (63), more recent OCA-B ChIP-seq data (19) suggest that, among the identified genes, *Lck*, *Cdc37*, *Kcnn4* and *S100a10* are direct OCA-B targets. In addition, a separate ChIP-seq analysis of genome-wide binding patterns of OCA-B, OCT2, and OCT1 in Ly7 DLBCL cells identified *BCL6*, encoding a master regulator of GC formation, as a direct target of these three factors. Notably, as discussed earlier, it was further shown that OCA-B, OCT2 and MEF2B form a ternary complex at the *BCL6* LCR to activate *BCL6* gene expression (18).

A more recent RNA-sequencing (RNA-seq) analysis has identified OCA-B–dependent transcriptomes in both *in vitro*-activated murine splenic B cells and human Burkitt’s lymphoma (Raji) cells (19). Many genes with known functions in GC reactions were shown to be OCA-B–dependent. For example, in the absence of OCA-B, genes required for GC formation and maintenance, such as *BCL6* and *MEF2b*, are downregulated, whereas genes related to post-GC differentiation, such as *IRF4, BCL2L1, NFKB1, NFKB2*, and *ZBTB32*, are upregulated (**Figure 1D**) (19). The ChIP-seq data from the same study demonstrated extensive colocalization of OCA-B with OCT1 and OCT2. For example, about 90% and 96% of the OCA-B peaks overlapped, respectively, OCT1 and OCT2 peaks. Short hairpin RNA knockdown experiments further demonstrated that OCA-B, OCT1, and OCT2 regulate each other and are all required for the proliferation of GC-derived lymphoma cells (19). Notably, ChIP-seq results also showed that, amongst other genes, *BCL6, MEF2b*, *IRF4, BCL2L1*, and *NFKB1* are direct OCA-B target genes – consistent with the presence of multiple OCA-B binding sites in their promoters and enhancers. Surprisingly, *IRF4* knockdown, but not ectopic expression of *BCL6*, rescued the lymphoma cell proliferation defect caused by OCA-B depletion. This result indicates that OCA-B controls a critical regulatory node in GC B cell proliferation.

It is also worth noting that OCA-B–regulated transcriptomes in Raji cells and in *in vitro*-activated splenic B cells are different. For example, *BCL6*, *CXCR4* and *XBP1* genes were identified as OCA-B–dependent genes in Raji cells but not in *in vitro*-stimulated B cells. More impressively, *IRF4* is upregulated in *OCA-B* knockdown Raji cells but downregulated in *in vitro*-stimulated *Oca-B* null B cells. Conversely, *AICDA* is downregulated in OCA-B-deficient Raji cells but upregulated in *in vitro*-stimulated *Oca-B* null B cells (19). These results likely reflect intrinsic differences between activated primary B cells and B lymphoma cells. As mentioned above, multiple direct OCA-B target genes with defined functions in GC B cells have been identified. Future investigations should clarify whether these genes mediate OCA-B function in GC responses.

## The function of OCA-B in follicular T helper cells

Follicular T helper (Tfh) cells are a subset of CD4^+^ T cells that play a critical role in germinal center responses and the establishment of long-term humoral immunity (64). The differentiation of Tfh cells depends on the transcription repressor BCL6 (65). In recent years, several independent studies have demonstrated a critical role for OCA-B in Tfh cell differentiation. Results from the initial report (8) showed high level expression of OCA-B in Tfh cells, as well as direct binding of OCA-B–OCT1/2 at the promoters of the *Bcl6* and *Btla* genes in these cells. Consistent with these results, the loss of OCA-B in mice led to a significant reduction in Tfh cell numbers, particularly in mesenteric lymph nodes (MLN) and Peyer’s patches (PP). OCA-B deficiency also led to a reduction in the BCL6 protein level in Tfh cells. Experiments using mixed bone marrow chimeras further demonstrated a CD4^+^ T cell-intrinsic defect in Tfh cell development in OCA-B–deficient mice (8). Data from a second study (66) showed that T cell-specific deletion of *Oca-B* is sufficient to cause defective GC formation and a decreased population of Tfh cells. Inactivation of *Oca-B* in T cells also resulted in an altered CD4^+^/CD8^+^ T cell ratio in the periphery and an increased number of regulatory T (Treg) cells. RNA-seq analysis of Tfh cells from immunized *Cd4Cre*, *Oca-B^fl/fl^* and control mice identified OCA-B–regulated genes in these cells. The most downregulated genes included *Irf4*, *Fasl*, *Ccr5*, *Irf8*, *ccl5*, and *Ccl22*, while the most upregulated genes included *Rab4a*, *Sox4*, *Kmt2a*, and *Kmt2d* (66). Data from a more recent third study (67) demonstrated that antigen-specific serum levels of IgG1, IgG2a, IgG2b and IgG3 are reduced in T cell-specific *Oca-B* conditional null mice. OCA-B was found to promote the reactivation of Tfh cells from the memory T cell pool. In a house dust mite (HDM)–induced bronchial asthma model, T cell-specific inactivation of *Oca-B* led to decreased serum levels of HDM-specific IgE and IgG1 antibodies, suggesting that OCA-B may represent a potential therapeutic target for asthma treatment In this more recent study, RNA-seq analysis further showed a significantly reduced expression of *Mef2b*, *Padi4*, and *S1pr2* and increased expression of *Sostdc1* and *Bhlhe40* genes in OCA-B–deficient Tfh cells (67). The discrepancy between the OCA-B–dependent genes identified in the second (66) and third (67) studies is likely due to differences in the cells used for RNA-seq analyses – total CD4^+^ T cells versus Tfh cells, respectively. As mentioned above, the initial report on the role of OCA-B in Tfh cell differentiation also showed that inactivation of OCA-B leads to a reduced BCL6 protein level in these cells – as well synergistic functions of OCA-B and OCT2 in activating the *Bcl6* promoter in a reporter assay (8). However, three subsequent studies, while confirming a role for OCA-B in Tfh cells, reported that OCA-B inactivation does not affect the *Bcl6* RNA level in these cells (66–68). Further experiments are needed to determine (i) whether, as suggested from the combined results, OCA-B can regulate the BCL6 protein level without affecting the *Bcl6* mRNA level in these cells and (ii) if that is the case, the underlying mechanism.

## The function of OCA-B in memory T cells

**Figure 2:**
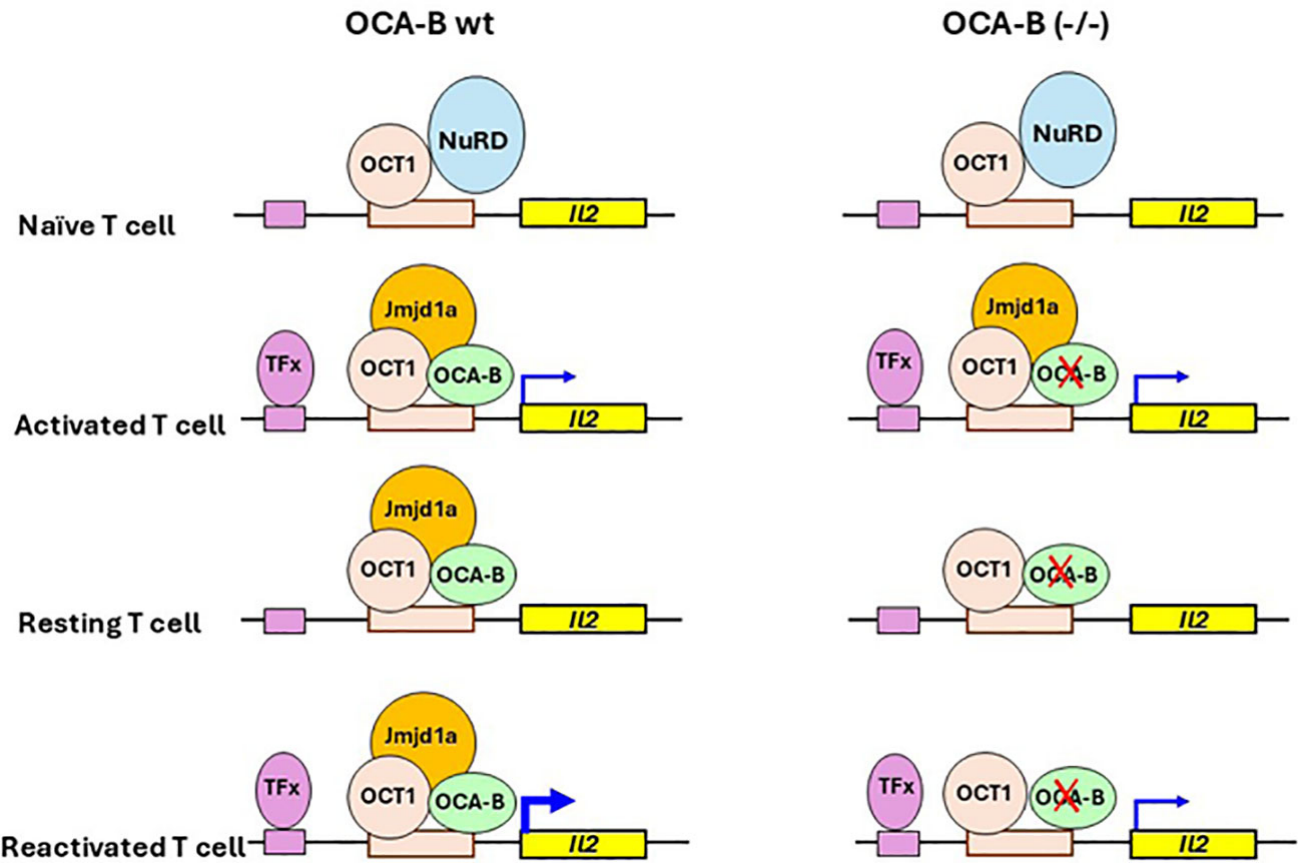
Model for OCA-B dependence for robust CD4^+^ T Cell memory recall response. In naive T cells, OCT1 interacts with the repressive NuRD complex that keeps the *Il2* gene inactive. The stimulation of T cells leads to NuRD dissociation from OCT1, which then recruits OCA-B and Jmjd1a to the *Il2* promoter. The resulting ternary complex, together with an unidentified transcription factor (TFx, one candidate is NFAT), activates the *Il2* gene. OCA-B is not required for Jmjd1a recruitment in the initial activation of *Il2* in the stimulated CD4^+^ T cells. In resting T cells, the OCT1–OCA-B–Jmjd1a complex remains bound at the *Il2* locus to keep it at a poised state for later robust expression, with OCA-B now being essential for maintaining Jmjd1a at the *Il2* locus. When resting T cells are stimulated again, TFx rebinds to the *Il2* locus and cooperates with the OCT1–OCA-B–Jmjd1a complex to induce rapid and robust expression of the *Il2* gene. The model is based on data reported in references 9 and 73. For further details and references, see the text.

Immune memory is a key component of adaptive immunity that enables the immune system to mount faster and stronger responses upon re-exposure to pathogens. Immune memory is primarily mediated by memory B cells, memory T cells, and plasma cells and their secreted antibodies (69). It has been reported that OCA-B and its associated transcription factor OCT1 are required for the generation of CD4^+^ T cell memory recall responses (9). While *Oct1* is constitutively expressed in CD4^+^ T cells (70), *Oca-B* is expressed at very low levels in thymocytes and naive CD4^+^ T cells but is induced upon antigen stimulation (71, 72). One important function of OCA-B in activated CD4^+^ T cells can be related to its regulation of the *Il2* gene, which encodes Interleukin 2, a key cytokine in stimulating the differentiation of antigen-activated T cells. The *Il2* gene is activated in naïve CD4^+^ T cells upon initial antigen stimulation and induced more rapidly and strongly during a second stimulation. In naïve CD4^+^ T cells, OCT1 is bound to the *Il2* gene locus in association with the repressive MTA2/NuRD complex. Upon a primary antigen stimulation, the Mta2/NuRD complex is dissociated from OCT1, which then interacts with newly expressed OCA-B to recruit histone3 lysine9 (H3K9) demethylase Jmjd1a to the *Il2* gene locus – thereby promoting robust *Il2* expression in restimulated T cells (**Figure 2**) (9, 73). Interestingly, the loss of OCA-B or OCT1 does not affect CD4^+^ T cell responses to the first stimulation but does lead to defective induction of a group of genes including *Il2, Il9, Il5, Csf2, Il10*, and *Ifng* etc. upon repeated stimulation *in vitro* (9). OCA-B–deficient T cells also show defective memory recall responses *in vivo* (9).

## The function of OCA-B in autoimmune diseases

Since persistent antigen stimulation is a common feature of autoimmunity, the above data imply a role for OCA-B in autoimmune diseases. In an *in vivo* study addressing this hypothesis, mice carrying *Oca-B* conditional alleles were bred to a non-obese diabetes-prone mouse strain (NOD) that develops spontaneous type I diabetes – an autoimmune disease resulting from the immune system attacking insulin-producing beta cells in the pancreas. Notably, data from this study showed that T cell-specific inactivation of OCA-B mediated by *Cd4Cre* can block the activation of autoreactive T cells in the pancreas and prevent the development of type 1 diabetes (22). A further analysis of NOD;*Cd4Cre Oca-B^fl/fl^* mice revealed that autoantigen-specific CD8+ T cells have reduced reactivity and islet penetration. It is worth noting that *OCA-B* prevents type I diabetes in an animal model-dependent manner (22). 

Complementary to the above results from genetic experiments, rationally designed membrane-penetrating OCA-B peptide inhibitors were found to reduce glucose levels, T cell infiltration, and proinflammatory cytokine expression in newly diabetic NOD mice (22). In a related study focusing on the role of OCA-B in memory T cells, it was shown that ectopic expression of the *Oca-B* gene enhances antiviral memory recall responses while having little effect on primary effector responses (74). Data from OCA-B mCherry reporter mice revealed a high *Oca-B* expression level in CD4^+^ central memory T cells. During the early stage of viral infection, *Oca-B* expression increases in T cell subsets containing memory precursor cells, which display a higher likelihood of survival and enhanced memory recall capacity (74). These results provide *in vivo* evidence for a critical function of OCA-B in memory recall responses in CD4^+^ T cells.

In addition to the potentially important role of OCA-B in type I diabetes as described above, OCA-B may also have a pathogenic function in multiple sclerosis (MS). Analysis of human multiple sclerosis (MS) databases reveals that CD4^+^ T cells from MS patients exhibit elevated OCA-B expression (75). In MS studies, the experimental autoimmune encephalomyelitis (EAE) model is widely used to replicate the autoimmune inflammation and demyelination seen in MS (76). In EAE models, mice are immunized with myelin or myelin peptides to activate antigen-specific CD4^+^ T cells that infiltrate the central nervous system to induce disease (66). In a chronic EAE mouse model (C57BL/6 background), T cell-specific deletion of *Oca-B* abolishes CNS T cell infiltration, proinflammatory cytokine production, and clinical symptoms. In a relapsing-remitting EAE model on the autoimmune-prone NOD background, OCA-B deficiency specifically protects mice against relapse (75). RNA-seq analyses of CNS-infiltrated T cells at remission and at peak relapse show that OCA-B promotes the expression of *Tcf7, Slamf6*, and *Sell* in proliferating CNS T cell populations during remission. At relapse, OCA-B deficiency leads to the accumulation of an immunomodulatory CD4^+^ T cell population expressing *Ccr9* and *Bach2*, along with a loss of proinflammatory gene expression from Th17 cells. These results suggest that OCA-B facilitates pathogenic Th17 cell differentiation (75). It should be noted that in the above autoimmune disease models, the T cells are exposed to antigens in a chronic manner and therefore can be characterized as memory-like T cells that are distinct from normal memory T cells. Results from these studies also provided proof of principle that OCA-B could be a drug target for the treatment of autoimmune diseases.

## The role of OCA-B in lymphomagenesis

During germinal center responses, GC B cells proliferate rapidly and undergo multiple rounds of somatic hypermutation, creating a high-risk environment for oncogenic mutations. Major forms of human B cell lymphomas such as Diffuse Large B-cell Lymphoma (DLBCL), Burkitt’s Lymphoma (BL), and Follicular Lymphoma (FL) all originate from the germinal center (77). Investigation of human B cell lymphoma samples showed that OCA-B is highly expressed in germinal center-derived lymphomas including DLBCL, BL and FL. In these tumors, high OCA-B expression levels are typically correlated with high expression levels of BCL6, which is a marker for GC-derived tumors (78). On the other hand, high OCA-B expression was not detected in B cell chronic lymphocytic leukemia (B-CLL), MALT-type lymphoma (with marginal zone B cell origin), and plasmacytoma samples (78). A genomic analysis has shown that the *OCA-B* gene, together with multiple other key GC regulatory genes such as *BCL6, PAX5 IRF8*, and *MYC*, are characterized by active super enhancers in human DLBCL cells (79). This observation suggests that the robust proliferation of DLBCL cells may be dependent upon an addiction to these genes. Functional analysis showed that knockdown of OCA-B in Ly1 DLBCL cells by short hairpin RNA (shRNA) significantly inhibited their growth, indicating an oncogenic role of OCA-B in B cell lymphomagenesis (79). In a more recent study, shRNA-mediated knockdown analyses in a panel of DLBCL cells demonstrated that OCT2 and OCA-B are both required for DLBCL cell growth (17). Furthermore, as mentioned above, the OCT2–OCA-B interaction is essential for DLBCL cell growth. *OCT2* or *OCA-B* mutations that disrupt this interaction inhibit proliferation of Ly10 cells (17), further supporting an oncogenic role of OCA-B in DLBCL and OCA-B as a potential drug target. In addition to its role in DLBCL, OCA-B has also been shown to be indispensable for growth of multiple myeloma (MM) cells (23). MM cells are also vulnerable to SMARCA2/4 degraders, suggesting that the mammalian SWI/SNF chromatin remodeling complex may be required for OCA-B–dependent MM cell growth (23). A recent study of OCA-B paralogs OCA-T1 and OCA-T2 has determined the crystal structures of OCT11–OCA-T1 and OCAT11–OCA-T2 in complexes with DNA (80). Further mutational scanning has uncovered mutation-sensitive hotspots and structurally constrained regions critical for the growth of a small cell lung cancer subtype that depends on OCT11–OCA-T1/2, suggesting OCT11–OCA-T1/2 as a drug target in treating this type of cancer (80), Given the structural similarity between OCA-B and OCA-T1/T2, the above findings further suggest OCA-B as a potential drug target for treating lymphomas with germinal center origin.

## Conclusion and perspective

Since the discovery of OCA-B over three decades ago, considerable progress has been made in understanding the mechanisms underlying OCA-B–dependent transcriptional activation and the function of OCA-B in B cell development, antigen-stimulated GC reactions, B cell lymphomagenesis, memory T cell function, and autoimmune diseases. However, several key issues remain to be addressed in future studies to achieve a more comprehensive understanding of the functions and associated mechanisms of action of OCA-B in these areas. (i) As OCA-B generally functions as a co-activator, the nature of its downstream effector functions through interactions with other components of the transcriptional machinery and chromatin modification factors remain to be fully elucidated, (ii) Given the critical cell-intrinsic functions of OCA-B in both GC B cells and Tfh cells during antigen-stimulated GC reactions, it will be important to identify the direct OCA-B target genes that mediate its function in these cell types. (iii) As the p35 isoform of OCA-B has a cytoplasmic function in stabilizing SYK and regulating the SYK-dependent signaling pathway, the extent to which the observed functions of OCA-B are attributable to cytoplasmic (p35) OCA-B vs nuclear (p34) OCA-B remains to be determined. (iv) Given the demonstrated addiction of DLBCL and BL cell lines to OCA-B, it will be important to test the effect of OCA-B inactivation in mouse lymphoma models to validate its oncogenic role in lymphoma progression. (v) Given the critical function of OCA-B in both normal GC reactions and GC B lymphomagenesis, it will be important to understand how expression of the *OCA-B* gene is regulated in GC B cells. Manipulating *OCA-B* expression offers a potential approach to suppress tumor progression and autoimmunity. (6) Given the function of OCA-B in memory T cells and in disease development in Type I diabetes and EAE models, it will be important to elucidate the direct OCA-B target genes that mediate OCA-B function in autoimmune models and to test OCA-B–dependence in other autoimmune disease models. Finally, given the essential role of OCA-B in GC reactions and its involvement in multiple pathologies, continued studies of OCA-B functions and mechanisms will further our understanding of germinal center biology and may guide the development of novel therapeutic strategies.
